# Automated Ventricular System Segmentation in Paediatric Patients Treated for Hydrocephalus Using Deep Learning Methods

**DOI:** 10.1155/2019/3059170

**Published:** 2019-07-07

**Authors:** Michał Klimont, Mateusz Flieger, Jacek Rzeszutek, Joanna Stachera, Aleksandra Zakrzewska, Katarzyna Jończyk-Potoczna

**Affiliations:** ^1^Department of Radiology, Poznań University of Medical Sciences, Poznań, Poland; ^2^Fast-Radiology, Poland; ^3^Practice, Poland; ^4^Department of Paediatric Radiology, Poznań University of Medical Sciences, Poznań, Poland

## Abstract

Hydrocephalus is a common neurological condition that can have traumatic ramifications and can be lethal without treatment. Nowadays, during therapy radiologists have to spend a vast amount of time assessing the volume of cerebrospinal fluid (CSF) by manual segmentation on Computed Tomography (CT) images. Further, some of the segmentations are prone to radiologist bias and high intraobserver variability. To improve this, researchers are exploring methods to automate the process, which would enable faster and more unbiased results. In this study, we propose the application of U-Net convolutional neural network in order to automatically segment CT brain scans for location of CSF. U-Net is a neural network that has proven to be successful for various interdisciplinary segmentation tasks. We optimised training using state of the art methods, including “1cycle” learning rate policy, transfer learning, generalized dice loss function, mixed float precision, self-attention, and data augmentation. Even though the study was performed using a limited amount of data (80 CT images), our experiment has shown near human-level performance. We managed to achieve a 0.917 mean dice score with 0.0352 standard deviation on cross validation across the training data and a 0.9506 mean dice score on a separate test set. To our knowledge, these results are better than any known method for CSF segmentation in hydrocephalic patients, and thus, it is promising for potential practical applications.

## 1. Introduction

### 1.1. Background

With an incidence of 1 in every 500 children [1], hydrocephalus is a common neurological condition. By definition, it is an increased amount of cerebrospinal fluid (CSF) in the ventricular system and/or subarachnoid space. There are multiple causes of hydrocephalus, from genetic disorders to traumas. Irrespectively of the cause, without treatment, this condition may be lethal, and the ramifications in treated cases range from infections caused by surgery to neurological disorders such as vision problems, epilepsy, neuroendocrine problems, and chronic headache.

**Figure 1:**
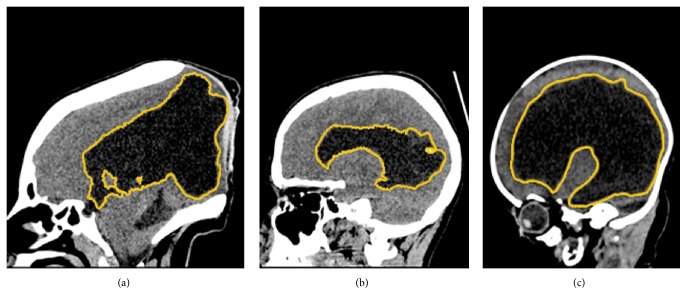
Variability in the size, shape, and distribution of CSF. Sagittal reconstructions from images of three different patients from our dataset that were treated for hydrocephalus. Examples include (a) a patient with encephalocele (treated surgically before the CT scan) and ((b) and (c)) patients with prematurity-associated intraventricular haemorrhage grade IV with bleeding extending into the brain tissue around the ventricles. Patients were treated with a ventriculoperitoneal shunt. Ventricular system boundaries are marked with a yellow line.

Treatment involves placement of a ventriculoperitoneal shunt or endoscopic ventriculostomy that enables outflow of excessive fluid. Regardless of the implemented method, patients have high readmission rates, which is related to surgical complications like shunt infection, as well as shunts malfunctions such as overdrainage, underdrainage, or obstruction. When the patient suffers neurological symptoms on readmission, a common strategy used by neurosurgeons is to assess the change in CSF volume. The cause of these symptoms might be due to an increase in volume, as well as a volume decrease from overdrainage. Usually, it is the radiologist who provides a description of the dynamics in CSF volume changes. When volume increases by a small amount, observation is the foundation of treatment. In contrast, a major rise in CSF volume requires surgical intervention. The reports from radiologists vary greatly in precision, depending on the methods of measurements. Objective methods for hydrocephalus diagnosis and monitoring include Evans' ratio, frontal and occipital horn ratio, and frontal horn radius [2], which are all methods of approximation of complex three-dimensional (3D) structures from measurements standardized in two dimensions. Ventricular system shapes may vary greatly, motivating the search for methods that do not make assumptions about the shape of the ventricles and measure the actual volume directly.  Figure 1 demonstrates the diversity in size, shape, and distribution of CSF within the ventricular system, using examples from our dataset. These differences are the consequence of different hydrocephalus manifestations and evolutions, but age-related anatomical differences have to be taken into account as well. Because of such a variable age group (0–18 years old), the differences in skull shape and size are also important factors that standard methods (based on selective measurements) fail to incorporate.

In recent years researchers used automated segmentation methods to address different medical problems, among them, coronary wall and atherosclerotic plaque segmentation [3], retinal vessel segmentation [4], brain segmentation [5], heart ventricle segmentation [6], and more generalized approaches, such as multiorgan segmentation [7, 8].

### 1.2. Related Work

To our knowledge, this is the first CSF segmentation attempt in hydrocephalic patients using deep learning techniques. Many other methods of physiological ventricular system segmentation have been proposed in the past [9–11]; unfortunately, there is no standardized dataset (such as ImageNet [12] for image classification tasks) that those methods could use as a benchmark. The first published methods were based on thresholding techniques which assumed that CSF is homogeneous in terms of radiodensity measured in Hounsfield units and were later joined by edge-detection, boundary-following methods, and a combination of the two [11]. While magnetic resonance imaging is the most popular choice among other authors [13, 14] we chose CT as an imaging modality because of its greater availability and lower cost. Most of the software providers [15, 16] for radiology departments provide some sort of semiautomated segmentation module, but the details of the underlying technology are not available; therefore, we were unable to compare to them.

CSF segmentation of patients without hydrocephalus was previously addressed by other authors, most recently by Chen et. al. [17]. They proposed architecture dedicated to CT images segmentation, which outperformed U-Net on their dataset. However, our work differs in terms of the addressed issues and analysed data. Hydrocephalus can manifest as an enlargement of numerous, often asymmetrical regions, whereas the physiological ventricular system maintains well-defined symmetry.

Other approaches include analysis of cranial ultrasound for ventricular system segmentation [18]; however, sonography and CT are image modalities that differ greatly. Therefore, we were not able to compare those results with ours.

### 1.3. Objective

The purpose of this study was to develop fully automated system that, given CT examination of the hydrocephalic patient, will calculate CSF volume within the ventricular system. An important modification was to create a system capable of comparing two examinations that yielded exact changes in volume between them.

## 2. Materials and Methods

### 2.1. Dataset Collection and Data Preprocessing

**Figure 2:**
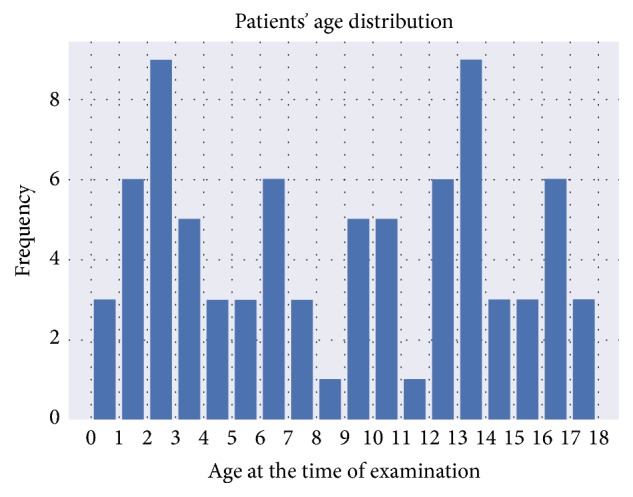
Patient age distribution in the dataset. This research included patients between 0 and 18 years of age. The most prevalent age in the dataset was between 2 and 3 years of age and between 13 and 14 years of age with nine examinations per group. The least represented groups were patients between 8 and 9 years of age and between 11 and 12 years of age with one examination in each group.

All CT scans were selected retrospectively from the department of radiology database at Karol Jonscher University Hospital, Poznan, Poland. Inclusion criteria consisted of patients aged between 0 and 18 years and either a new diagnosis of hydrocephalus or active treatment for this condition. We collected 80 CT scans from 63 patients. 46% of examinations were performed on female patients and 54% on male patients.  Figure 2 shows patient age distribution in the dataset. We analysed data as two-dimensional arrays; therefore, our data consisted of 19,443 2D images with approximately 240 images per examination.

In 43 CT scans, a low dose protocol was used, and in the remaining 37 a standard CT protocol was followed. Technical parameters of CT protocols are summarized in Table 1.

We randomly split our data into a training set containing 73 CT scans and a test set with the remaining 7 scans. The test set was kept separate for the whole process of training and refinement of our methods. It was used only once at the very end, after the training algorithm was used with optimal parameters.

For data segmentation, a 3D Slicer version 4.10 [19] was used. Each CT examination was segmented by radiologist in training and verified by radiology specialist with experience in paediatric hydrocephalus imaging. Segmentations and corresponding scans were stored as DICOM files. To facilitate data preparation, after obtaining 50 segmentations we trained a model that was used as a tool for preliminary segmentation. Those images were corrected afterwards by radiologist in training and verified by specialists in the same fashion as the first 50 examinations.

**Figure 3:**
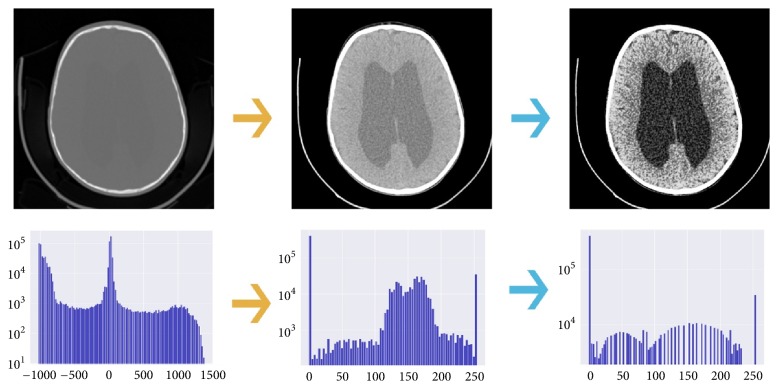
Two steps of preprocessing applied to every image in our dataset. Lower images show histograms at each state of the preprocessing (i.e., distribution of pixel values). First step (yellow arrows) consists of clipping pixel values outside -100 and 100 range and projecting those values to 0 to 255 array of integers. Second step (blue arrows) is a histogram equalization, a process of redistribution of most frequent intensity values on the image to increase global contrast.

Raw data was transformed to match the visual settings used by radiologists to assess the extent of hydrocephalus. Transformations consisted of clipping pixel values outside the range of -100 to 100 and projecting those values to 0 to 255 array of integer values and subsequently applying histogram equalization, a method used to increase the global contrast of the image. Clipping pixel values of the images to this range was chosen experimentally.  Figure 3 demonstrates preprocessing visually.

### 2.2. Algorithm Architecture and Training Process

**Figure 4:**
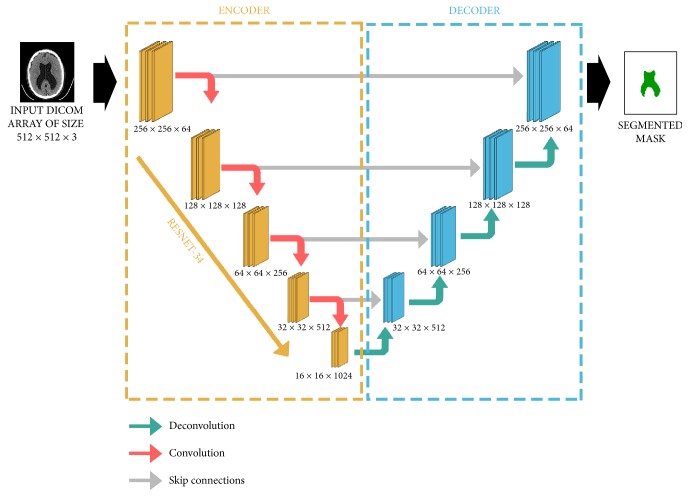
U-Net architecture consists of encoder and decoder steps. The encoder is based on ResNet34, which is the downsampling step. The decoder consists of symmetric layers that perform the upsampling step. The model uses skip-connection for better reconstruction of original image and prevention of vanishing gradient problem. The output is a probability matrix specifying whether given voxel is CSF.

U-Net, a network introduced by Ronnenberger et. al. [20], was our architecture of choice. It contains important features different from previous research approaches: downsampling (encoder) and upsampling (decoder), part of neural networks and so-called skip connections between those two. While introducing those novelties it was able to outperform state-of-the-art methods at the time of publication in biomedical data segmentation [20] and still remains the method of choice for many segmentation problems.  Figure 4 presents a conceptual architecture of U-Net.

In our modification of U-Net, ResNet34 [21] was used as an architecture for the encoder, and accordingly the decoder, which let us apply the idea of transfer learning. We initialized the encoder with weights learned on ImageNet dataset. This allowed the network to understand basic shapes, like edges and their composition [22]. As an upsampling method, we chose a pixel shuffle with subpixel convolution initialization [23] to reduce any checkerboard artefact effect. To improve regular convolution, a self-attention mechanism was used, which initially worked very well with Generative adversarial networks [24] and later proved to work with other architectures. The fastai library [25] was used for training, validation, and testing. The encoder (ResNet34) was trained using regular images with all three channels (RGB). As our data is a 512 by 512 pixel grayscale image, transfer learning was applied by copying the same image to all RGB channels. Neural networks were trained with batches of ten 2D images, which is the maximum we were able to fit into the GPU memory (12 GB) used. Hyperparameters of the network were chosen by running a series of experiments where their impact was analysed. During training, the “1cycle” [26] learning rate policy was utilized instead of a flat learning rate, which is an improved version of cyclical learning rates [27]. Half-precision training was also used, which allowed us to both accommodate bigger batches within the GPU memory and improve results. Another advantage of training with lower precision is that it may be easier to deploy trained model in the future. During the training process we used the Adam [28] optimization algorithm. To reduce the problem of unbalanced classes, the generalized dice loss [29] was applied as a loss function.  Table 2 summarizes hyperparameters along with other net parameters.

### 2.3. Postprocessing

We trained the model using 2D images. Predictions might contain inconsistencies because they were made one slice at the time, without knowing what was on the slice above and the slice below. That issue was addressed in postprocessing by removing or adding segmented pixels depending on the neighbour slice predictions. The algorithm was as follows:

1. All slices of an examination are predicted.
2. Each slice (except first and last ones) was processed by analysing its pixels and the pixels from the slice above and the slice below, according to precise rules:
  - If both pixels on the slices above and below have been segmented as CSF and the current slice's pixel was not, then it was relabelled as CSF.
  - If the opposite situation happened (i.e., both neighbours segmented as non-CSF, while current slice was labelled as CSF), then the pixel was relabelled to non-CSF.

An example of postprocessing is demonstrated as follows.

*Postprocessing Example on 4 × 4 Matrix. In this setting, “1” represents CSF*

(1) $$\begin{array}{c} \begin{array}{c} \text{Predictions }\begin{array}{c} \text{Slice below}\\ \left[\begin{array}{cccc} 0 & 0 & 0 & 0\\ 0 & 1 & 1 & 0\\ 0 & 0 & 1 & 0\\ 0 & 0 & 0 & 0 \end{array}\right] \end{array}\;\;\begin{array}{c} \text{Current slice}\\ \left[\begin{array}{cccc} 0 & 1 & 0 & 0\\ 0 & 0 & 1 & 0\\ 0 & 1 & 1 & 0\\ 0 & 0 & 1 & 0 \end{array}\right] \end{array}\;\;\begin{array}{c} \text{Slice above}\\ \left[\begin{array}{cccc} 0 & 0 & 0 & 0\\ 0 & 1 & 1 & 0\\ 0 & 0 & 0 & 0\\ 0 & 0 & 1 & 0 \end{array}\right] \end{array}\to \begin{array}{c} \text{Processed current slice}\\ \left[\begin{array}{cccc} 0 & 0 & 0 & 0\\ 0 & 1 & 1 & 0\\ 0 & 0 & 1 & 0\\ 0 & 0 & 1 & 0 \end{array}\right] \end{array} \end{array} \end{array}$$

### 2.4. Evaluation

The model performance was evaluated via 10-fold cross validation [30]. Our data contains patients that have more than one segmented examination (63 patients, 80 CT scans). To prevent overfitting and data leakage, scans were grouped via patients, not examinations. As the number of patients with multiple examinations was smaller than ten, each of those patients was first assigned to different folds and all the remaining patients were randomly sampled. This assured that each fold had a comparable number of examinations and patients. Details of the folds used can be found in Table 3.

For each of the folds, the model was trained with the exact same hyperparameters for four epochs using the training set. For evaluation, the following metrics were used: accuracy, dice, IOU (Intersection over Union, or the Jaccard Index), precision, recall, and volumetric similarity. Comprehensive explanations and comparison of these metrics can be found in [30]. Each metric was calculated on a single examination (3D image) and averaged between all patients in the fold. Those results were then aggregated using mean and standard deviation to show variability between folds.

## 3. Results

Detailed results of the 10-fold cross validation can be found in Table 4. With postprocessing, this fully automated method of segmentation achieved 0.9174 mean dice score with 0.0352 standard deviation (std). Applying postprocessing improved the results for dice, IOU, and precision metrics. The impact on other metrics was insignificant.

Detailed results of the test set evaluation can be found in Table 5. Mean dice score 0.9506 with 0.0276 standard deviation was achieved. Applying postprocessing improved dice, IOU, and precision metrics. The impact on other metrics was insignificant. The effect of postprocessing resembles the one in cross validation.

## 4. Discussion

While previously published methods concerning CSF segmentation were based on thresholding techniques, edge-detection, boundary-following methods, or a combination of these [11], they were based on a few assumptions. For example, thresholding, which assumes that CSF is homogeneous in terms of radiodensity as measured in terms of Hounsfield units, is unable to take into account differences between various CT scanners; therefore, five different research groups came up with five different cut-off values when exploring those methods [29]. Novel techniques of medical image segmentation include convolutional neural networks, which do not rely on small numbers of well-defined rules, but by definition have millions of parameters.

We propose a fully automated segmentation method that addresses specific clinical problem, i.e., monitoring outcomes in patients with hydrocephalus. This method, based on deep convolutional neural networks, uses two CT scans of the patient as the input and provides the answer to the question asked by paediatric neurosurgeons—did the volume of CSF increase, and if yes, by what amount? The motivation for our work comes from an observation that current methods face two fundamental problems. They are either very subjective (without any consistent approach) or time-consuming (manual work with a rigorous approach). Both of these are harmful to the quality results of patient examination and healthcare costs; therefore, faster and more objective solution are in high demand.

**Figure 5:**
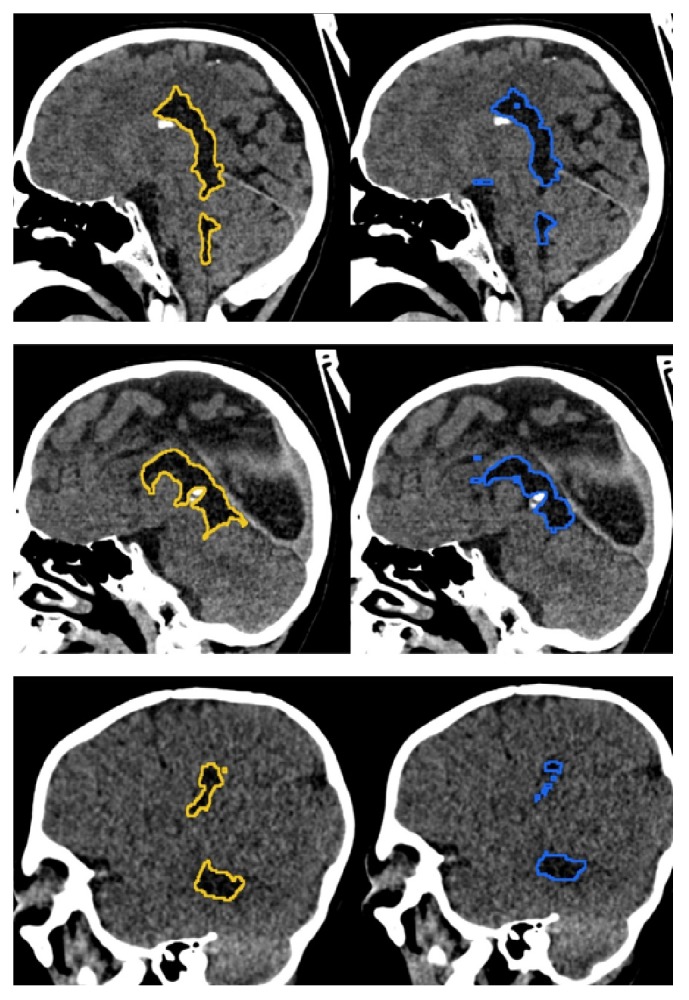
Examples of automatic segmentation (right column, blue line) and ground truth (left column, yellow line). Ground truth in our dataset is obtained by manual segmentation of ventricular system by radiologist in training and verification by the radiology specialist.

Code for this research was based on the fastai library [25], which offers ready-to-use innovative deep learning tools and algorithms. By applying such state-of-the-art deep learning methods for this task, human-like performance was achieved. However, exploration of other deep learning methods, especially 3D analysis, could further improve the results. Other researchers have reported improvements in segmentation scores when applying 3D analysis to their data [31]. Unfortunately, due to hardware restrictions, 3D analysis could not be performed at this stage of research.  Figure 5 demonstrates examples of mistakes made by automated segmentation on sagittal reconstruction of CT scans. A lack of 3D analysis of the examinations can be observed in discontinuity of CSF regions when visualized in sagittal plane (algorithm makes predictions on axial scans). Another potentially beneficial field of subsequent research would be increasing the number of CT scans analysed; however manual segmentation (which is crucial for data preparation) is time-consuming task.

All code used for this research is available on GitHub repository [32]. With the provided code, it is possible to reproduce the process of training on another dataset.

During the fine-tuning of the algorithm, many parameters were tested, and we think that sharing some of the paths that lead to worse performance would benefit the research community. We explored progressive resizing of input data during training, which consists of a training algorithm on the same dataset performed a few times, but with increasing resolution. An example of this approach would be training on 64 × 64 images, then 128 × 128, and finally 512 × 512 pixels data. Combining three consecutive CT slices as image channels was also tested, as it was presumed that it would provide more information about the 3D context of the image being segmented. None of these were showing significant improvements to the results, and some proved to be computationally more challenging.

Limitations of this study include potential bias in the algorithm performance due to small number of radiologists that performed segmentation tasks. Even though we validated our data carefully, there might be mistakes in our segmentation that we are not aware of, which the algorithm will reproduce. Another limitation is a derivative of our dataset. Hydrocephalus is a condition that affects also adults. Unfortunately our hospital is focused on treatment of paediatric patients; therefore, it was not evaluated on adult hydrocephalic patients due to the lack of data.

## 5. Conclusions

In summary, automated methods of CSF segmentation using deep learning state-of-the-art techniques were proven to work in highly diverse dataset of hydrocephalic, paediatric patients. With scores indicating near human-level performance, this method may be applied in a clinical setting as an aid to paediatric radiologists or neurosurgeons, providing a time-saving and reliable alternative to manual segmentation. To facilitate implementation in a clinical setting in other hospitals and to encourage further research in the field, we provide free access to all the code we produced for this research.

## Figures and Tables

**Table 1:** Low dose and standard dose CT scan protocols along with reconstruction parameters. All scans were acquired using a Siemens Somatom Definition AS.

*Low dose protocols* Average effective dose 1.85 mSv (std. 0.58 mSv)	For children over 1 but under 6 years of age	*Tube current:* Eff mAs CARE Dose4D *Tube potential:* 120 kV *Reconstruction algorithm:* Kernel C30s med. smooth FR *Reconstructed slice thickness:* 1.0 mm
	For children over 6 years of age	*Tube current:* 200 effective mAs *Tube potential:* 120 kV *Reconstruction algorithm:* Kernel H31f medium smooth + *Reconstructed slice thickness:* 1.0 mm

*Standard dose protocols* Average effective dose 3.16 mSv (std. 0.75 mSv)	For children under 6 years of age	*Tube current:* Eff mAs CARE Dose4D *Tube potential:* 120 kV *Reconstruction algorithm:* Kernel C30s med. smooth FR *Reconstructed slice thickness:* 1.0 mm
	For children over 6 but under 10 years of age	*Tube current:* Eff mAs 286 *Tube potential:* 120 kV *Reconstruction algorithm:* Kernel H30s medium smooth *Reconstructed slice thickness:* 1.0 mm
	For children over 10 years of age	*Tube current:* Eff mAs 343 *Tube potential:* 120 kV *Reconstruction algorithm:* Kernel H30s medium smooth + *Reconstructed slice thickness:* 1.0 mm

**Table 2:** Parameters of neural network architecture that were used consistently during training.

Network architecture		Training		Optimization	
Encoder	ResNet34	Learning rate	1e-4	Optimizer	Adam
Image size	512 x 512	Number of epochs	4	Learning rate policy	1cycle
Self-attention	True	Batch size	10	Loss	Generalized dice loss
Precision	FP16	Weight decay	1e-7		

**Table 3:** Folds used for cross-validation.

Fold	Patients in validation set	Number of examinations in validation set
0	P26,P31,P17,P40,P52,P25	6
1	P12,P33,P36,P20,P15,P39	7
2	P7,P28,P53,P34,P14,P41	7
3	P9,P56,P54,P24,P43,P47	7
4	P11,P13,P16,P21,P45,P38	7
5	P8,P27,P22,P6,P48	7
6	P3,P30,P55,P23,P35	7
7	P1,P4,P29,P49,P32	7
8	P5,P2,P37,P50,P51	8
9	P10,P19,P44,P46,P18	9

**Table 4:** Aggregated results for 10-fold cross validation.

	Without post-processing		With post-processing	
	Mean	Std	Mean	Std
Dice	0.9153	0.0351	*0.9174*	*0.0352*
IOU	0.8499	0.0553	0.8535	0.0557
Accuracy	0.9970	0.0016	0.9971	0.0016
Precision	0.9352	0.0236	0.9402	0.0213
Recall	0.9036	0.0445	0.9033	0.0462
Volumetric similarity	0.9644	0.0173	0.9637	0.0186

**Table 5:** Aggregated results for test set.

	Without post-processing		With post-processing	
	Mean	Std	Mean	Std
Dice	0.9482	0.0288	*0.9506*	*0.0276*
IOU	0.9027	0.0515	0.9069	0.0494
Accuracy	0.9969	0.0022	0.9970	0.0021
Precision	0.9433	0.0436	0.9463	0.0424
Recall	0.9549	0.0386	0.9566	0.0367
Volumetric similarity	0.9766	0.0223	0.9778	0.0218

## Data Availability

The CT scans used to support the findings of this study are available from the corresponding author upon request for researchers who are able to provide data anonymization framework implementing techniques, such as skull stripping [33]. Additional consent from the bioethical commission and Hospital board may be required. We provide GitHub repository with the code used in this project for possible improvements of our methods by other researchers. With the provided code, it is possible to reproduce the process of training on another dataset. Additionally, we plan to create a website where other researchers and radiologists may try our method on their own datasets. The link to it will be available on the GitHub repository.
